# Effect of combined pulmonary fibrosis and emphysema on patients with connective tissue diseases and systemic sclerosis: a systematic review and meta-analysis

**DOI:** 10.1186/s13075-021-02494-y

**Published:** 2021-04-06

**Authors:** Bon San Koo, Kyu Yong Park, Hyun Jung Lee, Hyun Jung Kim, Hyeong Sik Ahn, Shin-Young Yim, Jae-Bum Jun

**Affiliations:** 1 Department of Internal Medicine, Inje University Seoul Paik Hospital, Inje University College of Medicine, Seoul, South Korea; 2 Department of Physical Medicine and Rehabilitation, Ajou University School of Medicine, Suwon, South Korea; 3 Department of Physical Medicine and Rehabilitation, Jeju National University Hospital, Jeju, South Korea; 4 Graduate Program of Medicine, Ajou University Graduate School, Suwon, South Korea; 5 Department of Preventive Medicine, College of Medicine, Korea University, Seoul, South Korea; 6 Department of Rheumatology, Hanyang University Hospital for Rheumatic Diseases, Seoul, South Korea

**Keywords:** Pulmonary fibrosis, Emphysema, Interstitial lung diseases, Connective tissue diseases, Systematic review, Meta-analysis

## Abstract

**Background:**

This study aimed to analyze the literature systematically to determine the clinical characteristics and prognosis of patients with connective tissue disease (CTD) with combined pulmonary fibrosis and emphysema (CPFE) compared to those of patients with CTD-interstitial lung disease (CTD-ILD) without emphysema.

**Methods:**

We searched MEDLINE, EMBASE, Cochrane Library, and KoreaMed for relevant articles published before July 2019. Studies meeting all the following criteria were included: (1) original research studies evaluating the effect of CPFE on CTD, (2) studies that compared patients with CTD-CPFE to those with CTD-ILD without emphysema, and (3) studies providing data on physical capacity, pulmonary function, or death in patients with CTD. Clinical characteristics of patients with CTD-CPFE were compared with those of patients with CTD-ILD without emphysema, and the influence of CPFE on physical capacity, pulmonary function, and death was analyzed.

**Results:**

Six studies between 2013 and 2019 were included. Two hundred ninety-nine (29.5%) and 715 (70.5%) patients had CTD-CPFE and CTD-ILD without emphysema, respectively. Regarding the type of CTD, 711 (68.3%) patients had systemic sclerosis, 263 (25.3%) rheumatoid arthritis, and 67 (6.4%) other CTDs. Patients with CTD-CPFE had a higher frequency of pulmonary hypertension and pulmonary fibrosis > 20% of the total lung volume, higher ratio of the forced vital capacity to the diffusion capacity of the lung for carbon monoxide (DLCO), lower arterial oxygen pressure at rest, and lower DLCO compared to those in patients with CTD-ILD without emphysema. In addition, more deaths occurred among those with CTD-CPFE (odds ratio, 2.95; 95% confidence interval, 1.75–4.96).

**Conclusion:**

CTD-CPFE is associated with worse physical and pulmonary function and more deaths compared to those in CTD-ILD without emphysema. These findings indicate the need for increased awareness and close monitoring of patients with CTD-CPFE.

## Background

Interstitial lung disease (ILD) is characterized by fibrotic features generally in the lung bases of patients with connective tissue diseases (CTDs), such as rheumatoid arthritis (RA) and systemic sclerosis (SSc), observed on chest computed tomography (CT), high-resolution computed tomography (HRCT), and chest X-ray. Particularly, clinically significant ILD occurs in up to 40% of patients with SSc [1] and significantly increases the mortality rate [2]. As such, ILD has been recognized as an important clinical manifestation, specifically in the prognosis of patients with CTD. Recently, several studies reported that combined pulmonary fibrosis and emphysema (CPFE), as well as ILD, is a pulmonary manifestation within the spectrum of lung disease associated with CTD [3–8].

CPFE, which is characterized by the presence of both emphysema and interstitial pulmonary fibrosis, is a new disease; it exhibits distinct clinical, functional, radiological, and pathological characteristics [9]. The radiologic findings typically include upper-lobe centrilobular and/or paraseptal emphysema and a lower-lobe interstitial fibrotic pattern. In addition, most patients with CPFE have a mixed pattern of pulmonary function and marked reduction in the diffusion capacity of the lung for carbon monoxide (DLCO) [10]. Thus, complications such as acute lung injury, lung cancer, and pulmonary arterial hypertension may occur. The prevalence of pulmonary hypertension has been reported as 47–90%, which is much higher than the prevalence of chronic obstructive pulmonary disease (COPD) or idiopathic pulmonary fibrosis (IPF). Moreover, patients with CPFE-associated pulmonary hypertension have a poorer survival rate than those with COPD-associated or IPF-associated pulmonary hypertension [11–13]. Thus, CPFE in patients with CTD should also be recognized as an important pulmonary manifestation. However, the exact prevalence and clinical features are not well known.

Hence, in this systematic investigation, we aimed to determine the prevalence and characteristics of CPFE compared to those of ILD without emphysema in patients with CTD. In addition, by subgroup analysis, CPFE was compared with ILD with emphysema in patients with systemic sclerosis (SSc), which is one of the diseases of CTD.

## Methods

We searched MEDLINE, EMBASE, Cochrane Library, and KoreaMed for relevant articles published before July 2019. Studies were included in this systematic review if they met all of the following inclusion criteria: (1) original research studies evaluating the effect of CPFE on CTD, (2) studies that compared patients with CTD-CPFE to those with CTD-ILD without emphysema, and (3) studies providing data on physical capacity, pulmonary function, or death in patients with CTD. Studies with fewer than five cases, animal studies, review articles, and studies on patients aged ≤5 years were excluded.

Two investigators (KYP and SYY) independently performed data extraction using a predefined form. Any disagreement unresolved by discussion was reviewed by a third author (JBJ). The following variables were extracted from the studies: (1) demographic characteristics, including the first author’s name, year of publication, country where the study was performed, number of subjects, sex, and age; (2) type and diagnostic criteria for CTD; (3) the diagnostic method of CPFE and ILD without emphysema; (4) smoking history; (5) physical characteristics (such as the presence of anti-centromere antibodies, digital ulcers, and pulmonary hypertension), composite physiologic index, extent of pulmonary fibrosis, and partial pressure of arterial oxygen at rest; (6) pulmonary function test results and DLCO; (7) 6-min walk test results; and (8) the number of deaths.

The effect of CPFE on patients with CTD was analyzed in terms of physical capacity, pulmonary function, and death, and patients with CPFE were compared to those with ILD without emphysema. A subgroup analysis of the effects of CPFE was conducted for SSc because lung involvement in SSc is a major factor in the prognosis.

### Assessment of methodological quality

Two investigators (KYP and SYY) independently assessed the methodological quality of each study using the Newcastle-Ottawa Scale for assessing the risk of bias for non-randomized studies [14]. The Newcastle-Ottawa Scale is a tool used to assess the quality of non-randomized studies included in systematic reviews and/or meta-analyses. Each study was evaluated according to eight items, which were categorized as follows: selection of the study groups, comparability of the groups, and either the exposure or outcome of interest for case-control or cohort studies. The evaluation method included assigning stars to each study; the study with the highest quality may receive up to 10 stars. This method provides a quick visual assessment of the quality of a study. The score in the Newcastle-Ottawa Scale ranges from 0 to 10, where 10 indicates the highest methodological quality. Any discrepancies were addressed by a joint re-evaluation of the original article by a third author.

### Statistical analysis

The meta-analysis was performed using the Review Manager Software (RevMan version 5.3., Copenhagen: The Nordic Cochrane Centre, the Cochrane Collaboration, 2014). The pooled mean difference (MD; with 95% confidence interval [CI]) in physical capacity and pulmonary function between patients with CTD-CPFE and those with CTD-ILD without emphysema was calculated using the inverse-variance method. The pooled odds ratio (OR; with 95% CI) was also computed for sex; the number of smokers; the number of patients with anti-centromere antibodies, digital ulcers, and pulmonary hypertension; and the number of deaths for patients with CTD-CPFE and CTD-ILD without emphysema using the Mantel-Haenszel method.

We examined the heterogeneity across studies using the *I*^2^ statistic to quantify the percentage of variability that could be attributed to between-study differences. *I*^2^ values for all reports were calculated by the random effects model because of the inherent limitations of non-controlled studies. An *I*^2^ value > 50% was considered significantly heterogeneous. Statistical significance was defined as a *p* value < 0.05.

## Results

### Identification of studies and assessment of methodological quality

**Fig. 1:**
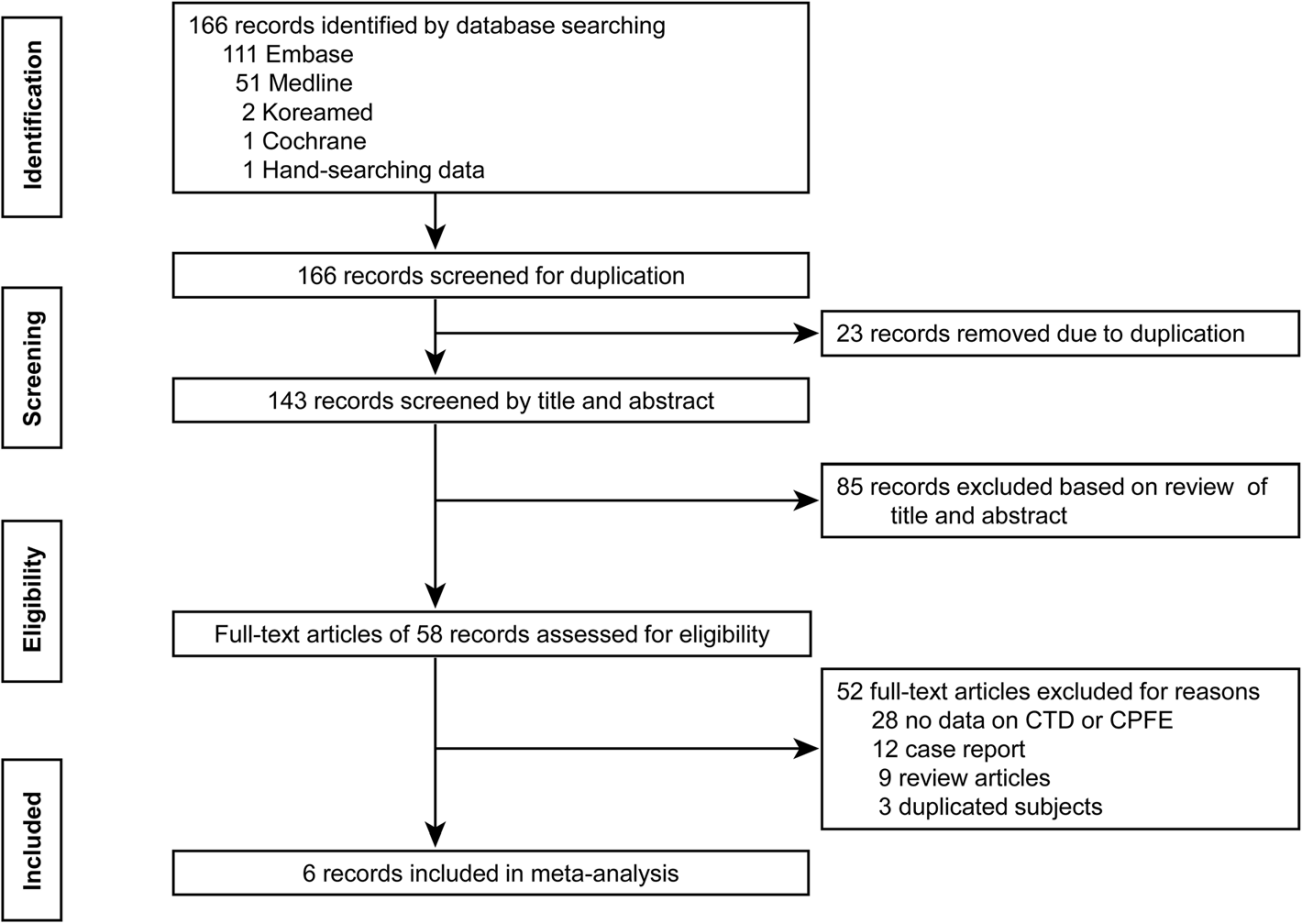
Flow diagram of the study selection. CTD, connective disease; CPFE, combined pulmonary fibrosis and emphysema

The study selection process is shown in Fig. 1. Among 346 records identified by the database search, six studies published between 2013 and 2019 were included in the analysis. Quality assessment of the six non-randomized studies was performed using the Newcastle-Ottawa Scale (Table 1). The methodological quality was scored as 8 in five non-randomized studies and as 3 in the remaining one study, which was a short report [14].

**Table 1:** Characteristics of studies included in the meta-analysis

Study/year	Country	Number of subjects with CTD (men/women)			Age of subjects (years; mean ± SD)		CTD	Diagnosis method of ILD	NOS score
		Total	CPFE	ILD without emphysema	CPFE	ILD without emphysema			
Antoniou et al./2016 [3]	UK	333 (74/259)	41 (18/23)	292 (56/236)	53.1 ± 15.1	54.4 ± 12.8	SSc	HRCT	8
Ariani et al./2019 [4]	Italy	239 (52/187)	43 (16/27)	196 (36/160)	57.7 ± 14.3	59.4 ± 13.49	SSc	CT	3
Champtiaux et al./2018 [5]	France	108 (40/68)	36 (27/9)	72 (13/59)	48.75 ± 17.02	46.75 ± 17.6	SSc	HRCT	8
Cottin et al./2011 [6]	France	68 (33/35)	34 (23/11)	34 (10/24)	57 ± 11	65 ± 10	RA 18 (53%) SSc 10 (29%) MCTD 2 (6%) Overlapping CTD 2 (6%) Sjogren’s syndrome 1 (3%) Polymyositis 1 (3%)	HRCT	8
Jacob et al./2018 [7]	South Korea and UK	245 (110/135)	129 (85/44)	116 (25/91)	63 (median)	62 (median)	RA	CT	8
Yamakawa et al./2018 [8]	Japan	21 (3/18)	16 (2/14)	5 (1/4)	60.5 ± 10.5	59.8 ± 11.7	SSc	HRCT	8
Total	–	1014 (312/702)	299 (171/128)	715 (141/574)	–	–	–	HRCT	8

*CTD* connective disease, *CPFE* connective disease and combined pulmonary fibrosis and emphysema, *ILD* interstitial lung disease, *NOS* Newcastle-Ottawa Scale, *SSs* systemic sclerosis, *RA* rheumatoid arthritis, *MCTD* mixed connective tissue disease, *CT* computed tomography, *HRCT* high-resolution computed tomography

### Study characteristics

The characteristics of the studies are shown in Table 1. A total of 1014 patients with CTD were included (312 [30.8%] men, 702 [69.2%] women). Two hundred ninety-nine (29.5%) patients had CTD-CPFE, and 715 (70.5%) had CTD-ILD without emphysema. Regarding the type of CTD, 745 (73.5%) patients had SSc, 263 (25.9%) RA, and 6 (0.6%) other CTDs. Among the six studies, four were for SSc [3–6], one for RA [7], and one for several CTDs [8]. SSc and RA were diagnosed based on the ACR-EULAR criteria [15–17], and ILD was confirmed by HRCT or CT.

### Prevalence of CPFE in patients with CTDs

CPFE prevalence was determined in three studies with a relatively large number of patients with SSc [3–5] (Table 2). In SSc-ILD with or without emphysema, the pooled prevalence of SSc-CPFE was 13.4% (94/703 patients). In a study of 276 patients with SSc, the CPFE prevalence regardless of the presence of ILD was 3.6%; moreover, CPFE was found in 17.4% of smokers and 5.3% of non-smokers [5].

**Table 2:** Prevalence of CPFE in patients with SSc or SSc-ILD

Study	Number of studied patients (*n*)			Prevalence (%, *n*)			
	SSc	SSc-ILD with or without emphysema	SSc-CPFE	CPFE in patients with SSc	SSc-CFPE in SSc-ILD with or without emphysema	SSc-CPFE in smokers	SSc-CPFE in non-smokers
Antoniou et al./2016 [3]	–	333	41	–	12.3	19.7 (26/132)	7.5 (15/201)
Ariani et al./2019 [4]	–	239	43	–	18.0	14.7 (16/109)	7.1 (25/351)
Champtiaux et al./2018 [5]	276	131*	10	3.6	7.6	–	–
Pooled data		703	94		13.4	17.4	5.3

*SSc* systemic sclerosis, *SSc-ILD* the patients with systemic sclerosis and interstitial lung disease, *CPFE* combined pulmonary fibrosis and emphysema, *CTD-CPFE* connective tissue disease and combined pulmonary fibrosis and emphysema, *CTD-ILD* connective tissue disease and interstitial lung disease

*Number of patients with confirmed ILD in patients with SSc

### Comparison of clinical features between CTD-CPFE and CTD-ILD without emphysema

The clinical characteristics of CTD-CPFE were compared to those of CTD-ILD without emphysema (Table 3). The CTD-CPFE group had a significantly more prominent smoking history than the CTD-ILD without emphysema group (six studies with ever-smokers: OR, 4.84; 95% CI, 3.52–6.65; two studies with current smokers: OR, 2.54; 95% CI, 1.24–5.19; and two studies with the amount of cigarettes smoked: MD, 13.64 pack-years; 95% CI, 7.77–19.48). In terms of physical and laboratory characteristics, the CTD-CPFE group had a higher number of patients with ACA and pulmonary hypertension, and lower arterial oxygen pressure at rest, than the CTD-ILD without emphysema group (two studies: OR, 0.81; 95% CI, 0.31–2.13; two studies: OR, 3.36; 95% CI, 1.85–6.09; and three studies: MD, − 0.89 kPa; 95% CI, − 1.26 to − 0.52). In terms of the pulmonary function test, the CTD-CPFE group had a higher forced vital capacity-to-DLCO ratio, lower DLCO, and lower transfer coefficient of the lung for carbon monoxide than the CTD-ILD without emphysema group (three studies: MD, 0.32; 95% CI, 0.15–0.48; six studies: MD, − 12.38%; 95% CI, − 15.62 to − 9.14; and three studies: MD, − 16.53; 95% CI, − 19.93 to − 13.13). In terms of the 6-min walk test, the CTD-CPFE group had a shorter walking distance than the CTD-ILD without emphysema group (two studies: MD, − 46.44 m; 95% CI, − 88.66 to − 4.22). Two studies reported 145 deaths (48.5%) among patients with CTD-CPFE and 121 deaths (16.9%) among those with CTD-ILD without emphysema; more deaths were recorded in patients with CTD-CPFE (two studies: OR of death, 2.95; 95% CI, 1.75–4.96).

**Table 3:** Meta-analysis on the effect of combined pulmonary fibrosis and emphysema (CPFE) on connective tissue disease (CTD), compared to interstitial lung disease (ILD) without emphysema

Characteristics	Number of patients (*n* = 1014)			Pooled mean difference or odds ratio [95% confidence interval]	Heterogeneity	
	Included study	CTD-CPFE (*n* = 299)	CTD-ILD without emphysema (*n* = 715)		*I*^2^ (%)	*P*
Age and gender						
Age of patients	5	170	599	0.23 [−2.44, 2.89]	0	0.50
Number of male patients	6	299	715	4.96 [3.60, 6.82]	60	0.03
Smoking history						
Number of ever-smokers	6	299	715	4.84 [3.52, 6.65]	74	0.002
Number of current smokers	2	77	230	2.54 [1.24, 5.19]	0	0.92
Amount of cigarette smoking (pack years)	2	70	106	13.63 [7.77, 19.48]	0	0.57
Physical and laboratory characteristics						
Number of patients with ACA	2	79	268	0.81 [0.31, 2.13]	0	0.89
Number of patients with digital ulcer	2	52	77	0.64 [0.29, 1.41]	0	0.77
Composite physiologic index	2	70	98	2.46 [−5.33, 10.24]	70	0.07
ILD extent, % of total lung volume	3	186	413	5.58 [−1.32, 12.47]	78	0.01
Number of patients with ILD extent > 20% of total lung volume	2	75	265	1.99 [1.18, 3.37]	22	0.26
Number of patients with pulmonary hypertension	2	73	361	3.36 [1.85, 6.09]	78	0.03
PaO_2_ at rest, kPa	3	107	382	−0.89 [−1.26, −0.52]	0	0.92
Pulmonary function test						
TLC, % of predicted	3	113	301	3.31 [−7.06, 13.69]	85	0.001
RV, % of predicted	2	70	104	10.55 [− 15.91, 37.01]	89	0.003
FEV1, % of predicted	4	240	512	−2.02 [−5.42, 1.37]	0	0.50
FVC, % of predicted	6	299	714	1.90 [−2.45, 6.26]	41	0.13
FEV1/FVC, % of predicted	3	86	109	−5.45 [−9.10, −1.80]	57	0.10
DLCO, % of predicted	6	299	707	−12.38 [− 15.62, −9.14]	36	0.17
FVC/DLCO	2	57	297	0.32 [0.15, 0.48]	0	0.35
KCO, % of predicted	3	199	214	−16.53 [−19.93, − 13.13]	0	0.63
6-minute walk test (6MWT)						
Walking distance in 6MWT, meters	2	60	76	−46.44 [−88.66, −4.22]	0	0.63
SPO_2_ after 6MWT, %	2	70	106	0.92 [−2.39, 4.23]	0	0.67
Decrease in SpO_2_ after 6MWT, %	2	60	74	−0.62 [−9.83, 8.59]	93	0.0002
Death						
Number of deaths	2	145	121	2.95 [1.75, 4.96]	0	0.78

*CTD-CPFE* connective tissue disease and combined pulmonary fibrosis and emphysema, *CTD-ILD* connective tissue disease and interstitial lung disease, *ACA* anti-centromere antibody, *ILD* interstitial lung disease, *PaO*_*2*_ arterial oxygen pressure at rest, *TLC* total lung capacity, *RV* reserve volume, *FEV1* forced expiratory volume in 1 s, *FVC* forced vital capacity, *DLCO* diffusing capacity of carbon monoxide, *KCO* carbon monoxide transfer coefficient, *SpO*_*2*_ peripheral capillary oxygen saturation

### Subgroup analysis of the effects of CPFE in patients with SSc

A meta-analysis on the clinical characteristics of the patients with SSc-CPFE is presented in Table 4; patients with SSc-CPFE were compared to those with SSc-ILD without emphysema. Subgroup analysis of age, sex, smoking history, physical characteristics, pulmonary function test results, and DLCO in patients with SSc-CPFE showed findings similar to those in patients with CTD-CPFE.

**Table 4:** Meta-analysis on clinical characteristics of the patients with systemic sclerosis (SSc) with combined pulmonary fibrosis and emphysema (CPFE) compared to the patients with SSc with interstitial lung disease (ILD) without emphysema

Characteristics	Number (*n* = 701)			Pooled mean difference or odds ratio [95% confidence interval]	Heterogeneity	
	Included study	SSc-CPFE (*n* = 136)	SSc-ILD without emphysema (*n* = 565)		*I*^2^ (%)	*P*
Age and gender						
Age of patients	4	136	565	−0.73 [−3.66, 2.19]	0	0.83
Number of male patients	4	136	565	3.99 [2.62, 6.06]	70	0.02
Smoking history						
Number of ever-smokers	4	136	565	3.16 [2.09, 4.78]	64	0.04
Physical characteristics						
Number of patients with ACA	2	79	268	0.81 [0.31, 2.13]	0	0.89
Number of patients with digital ulcer	2	52	77	0.64 [0.29, 1.41]	0	0.77
Composite physiologic index	2	70	98	2.46 [−5.33, 10.24]	70	0.07
ILD extent, % of total lung volume	2	57	297	7.02 [−4.53, 18.56]	85	0.009
Number of patients with ILD extent > 20% of total lung volume	2	75	265	1.99 [1.18, 3.37]	22	0.26
Number of patients with pulmonary hypertension	2	73	361	3.36 [1.85, 6.09]	78	0.03
PaO_2_ at rest, kPa	2	73	348	−0.94 [−1.41, −0.47]	0	0.84
Pulmonary function test						
TLC, % of predicted	2	79	267	−1.93 [−6.73, 2.88]	0	0.68
FEV1, % of predicted	2	77	362	−2.52 [−7.50, 2.45]	0	0.66
FVC, % of predicted	4	136	564	−0.65 [−4.96, 3.66]	0	0.58
FEV1/FVC, % of predicted	2	52	75	−4.62 [−9.41, 0.17]	72	0.06
DLCO, % of predicted	4	136	557	−14.31 [−17.31, −11.32]	0	0.41
FVC/DLCO	2	57	297	0.32 [0.15, 0.48]	0	0.35

*SSc-CPFE* systemic sclerosis and combined pulmonary fibrosis and emphysema, *SSc-ILD* sclerosis and interstitial lung disease, *ACA* anti-centromere antibody, *ILD* interstitial lung disease, *TLC* total lung capacity, *FEV1* forced expiratory volume in 1 s, *FVC* forced vital capacity, *DLCO* diffusing capacity of carbon monoxide

## Discussion

According to the results of our meta-analysis, patients with CTD-CPFE had different clinical features from those with CTD-ILD without emphysema. Patients with CTD-CPFE had higher rates of pulmonary hypertension and lower physical capacity compared to those with CTD-ILD without emphysema. Importantly, mortality was higher in patients with CTD-CPFE than in those with CTD-ILD without emphysema. In a subgroup analysis of SSc, similar results for the features of CPFE were found in the comparison of SSc-CPFE and SSc-ILD without emphysema. Therefore, the prognosis may be poorer with the presence of CPFE as opposed to pulmonary fibrosis without emphysema in patients with CTD. Notably, a recent CPFE study that included some patients with CTD showed that patients with less fibrosis at baseline (< 5%) had a better prognosis, suggesting that fibrosis has a significant influence on the prognosis [18].

Cottin et al. showed that the smoking history and pulmonary function profile were similar between smoking-related CPFE and CTD-related CPFE. Nevertheless, patients with CTD-related CPFE had a better prognosis compared to those with smoking-related CPFE, and those with CTD-ILD without emphysema had a better prognosis than those with IPF [6]. In the current meta-analysis, we focused on the comparison between CPFE and ILD without emphysema in patients with CTD. Regarding the poorer prognosis for CPFE than for ILD without emphysema in patients with CTD, the presence of emphysema might be related to the risk factors in patients with ILD [19].

In SSc, ILD is one of the most common complications and the most common cause of death [20, 21]. The prevalence of CPFE has been reported as 3.6% for SSc and 13.4% for SSc-ILD with or without emphysema [3–5], indicating that some cases are overlooked when evaluating the lung involvement in SSc. Systematic classification and prognosis-related factors for CTD-CPFE as well as idiopathic CPFE should be studied in the future.

Yamakawa et al. conducted a surgical lung biopsy study in 21 patients with SSc-ILD [8]. Pathological pulmonary emphysema was seen in 16 (76.2%) patients, of whom 62.5% were never-smokers. On HRCT, an interstitial abnormality with an area of low attenuation was seen in 31.3% of patients, and this was reported as a novel and radiopathological feature specific to SSc-ILD. However, as the authors pointed out, in the definition of existing CPFE, it should be understood that the pathological pulmonary emphysema shown in the current study and emphysema that appears radiologically predominant in the upper poles on HRCT in other studies have different characteristics. In addition, paraseptal emphysema, which can often be seen in non-smokers, is different from centrilobular emphysema, which is caused by exposure to smoking, on HRCT. These evidences suggest that CPFE in patients with CTD is associated with an immunological mechanism other than the commonly known emphysema.

In our results, the prevalence of CPFE in smokers was 17%, which shows that smoking is a major risk factor for CPFE. However, in non-smokers, the prevalence was 5.3%, suggesting another possible risk factor for CPFE. Studies on RA-ILD conducted in Korea and the UK showed a high CPFE prevalence (27%) in never-smokers [7] and suggested that CPFE is independently associated with a worsened outcome and a definite usual interstitial pneumonia pattern on CT. Therefore, patients with autoimmune diseases, such as SSc, should be carefully monitored for emphysema regardless of smoking using low-dose HRCT and the pulmonary function test, especially for those with ILD. In addition, it is necessary to develop new biomarkers or modalities that can differentiate CPFE and quantify risk.

Moreover, ILD in SSc is more often accompanied by emphysema; thus, the high CPFE prevalence in ILD suggests that ILD itself may be a risk factor for the development of emphysema and that CPFE may be a pulmonary manifestation within the spectrum of CTD-ILD [22]. Interestingly, while smoking is a major risk factor for CPFE, Jacob et al. suggested that the prevalence of emphysema is also high in never-smokers [7]. The mechanism of CPFE should be further clarified in future studies, although several possibilities have been suggested [3, 5, 23].

Our study, which is the first meta-analysis of the effect of CPFE on CTD, has some limitations. First, the small number of studies included in this meta-analysis limits the strength of the evidence regarding the effect of CPFE on the clinical manifestations of CTD. Second, we included various types of CTDs, including extremely rare diseases, in the meta-analysis. While we have conducted a subgroup analysis of SSc, a subgroup analysis of other CTDs with different characteristics, such as RA, is needed. Third, the extent of lesions in ILD and CPFE can vary significantly from patient to patient, when interpreting the results and prognosis.

## Conclusions

Patients with CTD-CPFE showed greater physical and pulmonary function deterioration, decreased exercise capacity, and increased mortality compared to patients with CTD-ILD without emphysema. Although the prevalence of CTD-CPFE is lower than that of CTD-ILD without emphysema, CPFE should be carefully evaluated using low-dose HRCT and the pulmonary function test.

## Supplementary Information

**Additional file 1.** Search strategy: MEDLINE, EMBASE, Cochrane, and KoreaMed.

## Data Availability

All data generated or analyzed during this study are included in this published article.

## Abbreviations

CTD: Connective tissue disease
CPFE: Combined pulmonary fibrosis and emphysema
ILD: Interstitial lung disease
RA: Rheumatoid arthritis
SSc: Systemic sclerosis
COPD: Chronic obstructive pulmonary disease
MD: Mean difference
CI: Confidence interval
OR: Odds ratio
HRCT: High-resolution computed tomography
DLCO: Diffusing capacity of the lung for carbon monoxide
