# Supplementary material for: Effect of combined pulmonary fibrosis and emphysema on patients with connective tissue diseases and systemic sclerosis: a systematic review and meta-analysis
Source: Arthritis Res Ther. 2021 Apr 6;23:100. doi: 10.1186/s13075-021-02494-y (PMC8022385; doi:10.1186/s13075-021-02494-y)
Supplement: Supplementary file 1 — Additional file 1. Search strategy: MEDLINE, EMBASE, Cochrane, and KoreaMed. [file 13075_2021_2494_MOESM1_ESM.doc]

**Supplementary Material**

Search strategy: MEDLINE

| 1 | Pulmonary fibrosis [MeSH Terms] | 23,434 |
| --- | --- | --- |
| 2 | Emphysema [MeSH Terms] | 27,714 |
| 3 | #1 AND #2 [MeSH Terms] | 1,124 |
| 4 | (Combined pulmonary fibrosis [Title/Abstract] AND emphysema [Title/Abstract]) | 236 |
| 5 | (Combined pulmonary fibrosis[Text Word] AND emphysema[Text Word]) | 236 |
| 6 | CPFE[Text Word] | 335 |
| 7 | #4 OR #5 OR #6 | 414 |
| 8 | #3 OR #4 OR #5 OR #6 | 1,385 |
| 9 | Systemic sclerosis[MeSH Terms] | 283 |
| 10 | Systemic sclerosis[Title/Abstract] | 14,906 |
| 11 | Systemic sclerosis[Text Word] | 14,908 |
| 12 | #9 OR #10 OR #11 | 24,273 |
| 13 | Rheumatoid arthritis[MeSH Terms] | 112,745 |
| 14 | Rheumatoid arthritis[Title/Abstract] | 105,759 |
| 15 | Rheumatoid arthritis[Text Word] | 105,764 |
| 16 | RA[Text Word] | 75,979 |
| 17 | #13 OR #14 OR #15 OR #16 | 178,782 |
| 18 | Mixed connective tissue disease[MeSH Terms] | 1,636 |
| 19 | Mixed connective tissue disease*[Title/Abstract] | 2,002 |
| 20 | Mixed connective tissue disease*[Text Word] | 2,497 |
| 21 | MCTD[Text Word] | 948 |
| 22 | #18 OR #19 OR #20 OR #21 | 2,592 |
| 23 | Dermatomyositis[MeSH Terms] | 7,870 |
| 24 | Dermatomyositis[Title/Abstract] | 9,131 |
| 25 | Dermatomyositis[Text Word] | 10,762 |
| 26 | #23 OR #24 OR #25 | 10,762 |
| 27 | Polymyositis[MeSH Terms] | 9,279 |
| 28 | Polymyositis[Title/Abstract] | 5,934 |
| 29 | Polymyositis[Text Word] | 6,360 |
| 30 | #27 OR #28 OR #29 | 12,336 |
| 31 | Systemic lupus erythematosus[MeSH Terms] | 59,436 |
| 32 | Systemic lupus erythematosus[Title/Abstract] | 50,353 |
| 33 | Systemic lupus erythematosus[Text Word] | 50,363 |
| 34 | #31 OR #32 OR #33 | 72,523 |
| 35 | #12 OR #17 OR #22 OR #26 OR #30 OR #34 | 271,751 |
| 36 | #8 AND #35 | 51 |

Search strategy: EMBASE

| 1 | Pulmonary fibrosis.af. | 30,174 |
| --- | --- | --- |
| 2 | Emphysema.af. | 43,638 |
| 3 | 1 and 2 | 1,849 |
| 4 | (Combined pulmonary fibrosis and emphysema).af. | 500 |
| 5 | 3 or 4 | 1,849 |
| 6 | Systemic sclerosis.af. | 31,301 |
| 7 | Rheumatoid arthritis.af. | 216,617 |
| 8 | Mixed connective tissue disease.af. | 4,322 |
| 9 | Dermatomyositis.af. | 16,663 |
| 10 | Polymyositis.af. | 10,985 |
| 11 | Systemic lupus erythematosus.af. | 99,293 |
| 12 | #6 OR #7 OR #8 OR #9 OR #10 OR #11 | 337,612 |
| 13 | #5 AND #12 | 111 |

Search strategy: Cochrane

| 1 | MeSH descriptor: [Pulmonary Fibrosis] explode all trees | 516 |
| --- | --- | --- |
| 2 | MeSH descriptor: [Emphysema] explode all trees | 237 |
| 3 | #1 and #2 (Word variations have been searched) | 7 |
| 4 | (Combined pulmonary fibrosis and emphysema):ti,ab,kw (Word variations have been searched) | 25 |
| 5 | #3 OR #4 (Word variations have been searched) | 29 |
| 6 | MeSH descriptor: [Scleroderma, Systemic] explode all trees | 538 |
| 7 | ("Systemic sclerosis"):ti,ab,kw (Word variations have been searched) | 1,096 |
| 8 | #6 OR #7 (Word variations have been searched) | 1,211 |
| 9 | MeSH descriptor: [Arthritis, Rheumatoid] explode all trees | 6,047 |
| 10 | (Rheumatoid arthriti):ti,ab,kw (Word variations have been searched) | 15,322 |
| 11 | #9 OR #10 | 15,598 |
| 12 | MeSH descriptor: [Mixed Connective Tissue Disease] explode all trees | 3 |
| 13 | (Mixed connective tissue disease*):ti,ab,kw (Word variations have been searched) | 132 |
| 14 | #12 OR #13 | 132 |
| 15 | MeSH descriptor: [Dermatomyositis] explode all trees | 87 |
| 16 | (Dermatomyositis):ti,ab,kw (Word variations have been searched) | 295 |
| 17 | #15 OR #16 (Word variations have been searched) | 295 |
| 18 | MeSH descriptor: [Polymyositis] explode all trees | 93 |
| 19 | (Polymyositis):ti,ab,kw (Word variations have been searched) | 177 |
| 20 | #18 OR #19 | 223 |
| 21 | MeSH descriptor: [Lupus Erythematosus, Systemic] explode all trees | 1,021 |
| 22 | ("Systemic lupus erythematosus"):ti,ab,kw (Word variations have been searched) | 1,870 |
| 23 | #21 OR #22 (Word variations have been searched) | 2,185 |
| 24 | #8 OR #11 OR #14 OR #17 OR #20 OR #23 (Word variations have been searched) | 18,940 |
| 25 | #5 AND #24 (Word variations have been searched) | 1 |

Search strategy: KoreaMed

| 1 | ((Pulmonary Fibrosis[All])) AND (Emphysema[All]) | 22 |
| --- | --- | --- |
| 2 | ((((((systemic sclerosis[All])) OR (rheumatoid arthritis[All])) OR (Mixed Connective Tissue Disease[All])) OR (Dermatomyositis[All])) OR (Polymyositis[All])) OR (systemic lupus erythematosus[All]) | 2,945 |
| 3 | #1 AND #2 | 2 |
